# Substrate-induced hybridization of plasmon modes in the composite nanostructure of nanodisk array/thin film for spectrum modulation

**DOI:** 10.1515/nanoph-2024-0159

**Published:** 2024-08-02

**Authors:** Yuzhang Liang, Shuwen Chu, Xinran Wei, Haonan Wei, Sun Cheng, Yi Han, Wei Peng

**Affiliations:** School of Physics, 12399 Dalian University of Technology, Dalian 116024, China; College of Physical Science and Technology, Dalian University, Dalian 116622, China; Department of Anaesthesia, Second Hospital of Shanxi Medical University, Taiyuan 030001, China

**Keywords:** plasmonic nanostructure, hybridization coupling, substrate-induced coupling, spectral modulation, biosensor

## Abstract

Hybridization coupling among plasmon modes is an effective approach to manipulate near-field properties thus optical spectral shapes of plasmonic nanostructures. Generally, mode hybridization coupling is achieved by modifying the topography and dimensions of nanostructures themselves, with few concerns about substrate-induced manipulation. Herein, we propose a composite nanostructure consisting of a gold (Au) nanodisk array and a thin Au film supported by a dielectric substrate. In this configuration, both the refractive index of the dielectric substrate and thin gold film’s thickness mediate the interaction of plasmon modes supported by upper and lower interfaces of the composite nanostructure, resulting in two hybridized plasmon modes. We systematically investigate the relationship between optical fields at the top surface of plasmon modes before and after the hybridization coupling. Specifically, the near-field amplitude at the top surface of the unhybridized modes is stronger than that of individual hybridized mode, and lower than the near-field summation of these two hybridized modes. This work not only provides a straightforward strategy for generating two plasmon modes in a nanostructure but also elucidates the variation of the optical field during the hybridization process, which is of crucial significance for applications, such as upconversion enhancement and multi-resonance sensing.

## 1 Introduction

Metallic nanostructures have attracted tremendous attention across a wide range of emerging applications, from high-sensitivity biochemical detection to high-performance integrated optoelectronic devices [1], [2]. Most of these applications benefit significantly from their unique optical near-field properties of substantial enhancement and nanometric confinement. Highly enhanced local electromagnetic fields strongly depend on their geometries and sizes [3], [4], [5]. An effective strategy to tailor near-field properties and optical spectral lineshape of metallic nanostructures is to employ the coupling and hybridization among plasmon modes [6], [7], [8]. Currently, a wide variety of methods have been developed to manipulate the coupling of plasmon modes and their optical spectra. The most classical approach is to exploit complex nanostructures comprising multiple closely spaced nanoantennas, where the strong interaction of multiple plasmon modes forms new bonding and antibonding hybrid modes [9], [10], [11]. This interaction directly determines the near-field profiles and optical spectral lineshape, such as closely spaced nanodisk cluster [12], [13], concentric disk/ring nanocavity [14], [15], three-layer gap plasmonic nanocavity [16], [17], [18], [19], and the like [20]. The highly localized near-fields from strong coupling in complex nanostructures are particularly suitable for improving sensing performance. Another common profound method to modulate optical spectrum is by breaking the symmetry of plasmonic nanostructure to excite Fano resonance with an asymmetric narrowband lineshape [21], [22], [23] through the strong coupling among plasmon modes, Examples include asymmetric ring/disk structure [24], [25], symmetry-broken hybrid nanoshells [26], *etc*.

Recently, a facile spectral modulation strategy for plasmonic nanostructures has been introduced by regulating the symmetry of ambient surroundings around the nanostructure [27]. Specifically, when an individual nanostructure or a planar nanostructure array, such as, a single nanosphere, nanocube [11], nanohole/nanodisk array [28], *etc*., is placed onto a bulk and plane dielectric substrate, an asymmetric environment is formed due to different refractive index (RI) between the ambient environment and the dielectric substrate. This asymmetry results in the generation of new hybrid plasmon mode and complex spectrum. Unfortunately, the substrate-induced hybrid plasmon mode tends to concentrate the enhanced electric field inside the high RI substrate, reducing the near-filed enhancement of the original plasmon mode in the ambient medium. To address this issue, some practical approaches have been proposed, such as using a water-index-matched Cytop film as the substrate, employing an opaque metallic film, lifting a nanostructure with high dielectric nanopillar, and the like [29], [30], [31]. Among these methods, using an opaque metallic film as a supporting substrate can greatly enhance near-field around the nanostructure compared to its individual counterpart, which is beneficial to high-sensitivity biomolecular detection. However, this method cancels the ability to adjust the spectral properties via the dielectric substrate by shielding its influence [32]. Therefore, it is highly desired that a new design strategy for plasmonic nanostructures can eliminate the negative effect of the dielectric substrate on the near-field enhancement while preserving the ability to regulate the spectrum using the dielectric substrate.

In this paper, we propose theoretically and fabricate experimentally a facile and large-scale plasmonic composite nanostructure composed of a quasi-orderly triangular nanodisk array and a semi-transparent Au film onto a bulk dielectric substrate. In this plasmonic composite nanostructure, two resonance modes are observed at normal incidence, wavelength positions of which are simultaneously tailored by both the RI of the dielectric substrate and the thickness of translucent Au film, particularly long wavelength resonance mode. The generation of these two resonance modes originates from the hybridization coupling of plasmon modes at the upper and lower surfaces of the composite nanostructure. The optical field variation among resonance modes before and after hybridization is further revealed through both bulk RI sensitivity and molecular detection capability. This work proposes a simple strategy to modulate the optical spectrum and near-field distribution of plasmonic nanostructure by utilizing substrate-induced hybridization, opening new avenues for designing novel optoelectronic devices.

## 2 Materials and methods

### 2.1 Materials

11-mercaptoundecanic acid (MUA), 1-(3-Dimethylamino-propyl)-3-ethylcarbodiimide hydro-chloride (EDC), and N-Hydroxysuccinimide (NHS) are from J&K Scientific; Phosphate buffer saline (PBS, pH 7.4) and bovine albumin (BSA) are purchased from Shanghai Sheng Gong; Concanavalin A (Con A) and Ribonuclease B (RNase B) are acquired from Sigma-Aldrich; All glassware used are thoroughly cleaned with aqua regia (a mixture consisting HCl and HNO_3_ in a 3:1 ratio), rinsed thoroughly with ultrapure water and oven-dried before use. All chemical reagents are of analytical grade.

### 2.2 Fabrication of the composite nanostructure

The proposed composite nanostructure is fabricated via the combination of template transfer nanoprinting and vacuum coating technology. The detailed fabrication procedures are summarized as follows: firstly, the dielectric substrates of quartz, K9, Al_2_O_3_, and SF11 are sequentially washed in an ultrasonic cleaner with ethanol, acetone, ultrapure water for 5 min then dried with a stream of nitrogen; Secondly, a certain thickness of Au film is deposited on the prepared dielectric substrate by using magnetron sputtering coater. The coated substrate then is stored in a sealed and drying container for standby. The thickness of Au film is controlled by the sputtering time. Thirdly, an ultrathin aluminum oxide (AAO) membrane with a highly ordered triangular nanohole array is prepared through a two-step anodization method. The prepared ultrathin AAO membrane has a quasi-lattice constant of ∼450 nm, a thickness of ∼350 nm, and nanohole diameter randomly varying from 260 nm to 360 nm, as reported in our previous work [33]. Afterward, the ultrathin AAO membrane is transferred onto the dielectric substrate covered with a translucent Au film. Finally, a 40-nm-thick Au film is deposited, followed by the removal of the AAO membrane with epoxy adhesive tape to form the proposed composite nanostructure of an Au nanodisk array/thin film.

### 2.3 Optical characterization

The reflection spectrum and sensing response of the proposed composite nanostructure are characterized by using the home-built measured system. This system includes a broadband light source (HL-2000, Ocean Optics Inc.) and a miniature fiber-optic spectrometer (AvaSpec-MINI4096CL, Avantes Inc.). These components are connected to two branch ends of a bifurcated optical fiber jumper, with its common end connected to an optical fiber collimator. The large-scale composite nanostructure is integrated with a polydimethylsiloxane (PDMS) flow cell, with inlet and outlet holes connected to the tubing. A peristaltic pump is used to inject sample solution at a constant flow rate of 0.5 mL/min. In addition, real-time data acquisition could be processed by a custom-written LabVIEW program.

### 2.4 Numerical simulations

Numerical simulations of the proposed composite nanostructure are performed using finite-difference time-domain (FDTD) method-based FDTD Solution software to obtain its reflection spectrum. In this simulation, anti-symmetric and symmetric boundary conditions are adopted along the *x* and *y* directions to accelerate calculation speed and save computational memory. Meanwhile, perfectly matched layers boundary condition is set at the *z* direction. To obtain convergent and accurate results, a refined mesh of 2 nm × 3.46 nm × 2 nm is used at the Au region. The polarization and propagation directions of the incidence light are along the *x* and *z* directions, respectively. We only consider the periodic plasmonic nanodisk array and ignore the randomized distribution of nanodisk diameter. Thereby, the periodicity, diameter, and thickness of the nanodisk are set as 450 nm, 260 nm, and 40 nm, respectively. The permittivity of Au in the visible and near-infrared range is described by fittting the measured data of Johnson and Christy [34]. The RIs of dielectric substrates for quartz, K9, Al_2_O_3_, and SF11 are specified as constants of 1.46, 1.513, 1.732, and 1.785, respectively. Notably, three-dimensional electric field distributions of the resonance mode are obtained using finite element-based COMSOL Multiphysics software, which demonstrates more details of the spatial distribution of the electromagnetic field, including multiple cross-section planes along different directions.

## 3 Results and discussions

**Figure 1:**
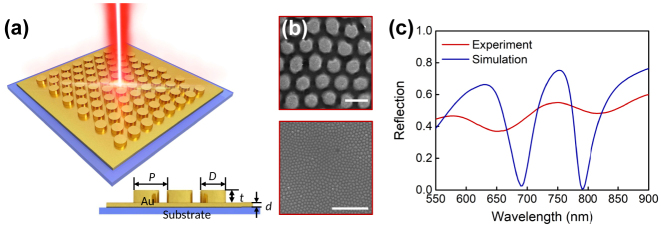
Substrate-modulated plasmonic composite nanostructure and its typical optical spectrum. (a) Three-dimensional schematic diagram and its cross-section view with all geometric parameters indicated. (b) The SEM images of the fabricated sample. Scale bar: 500 nm and 10 um. (c) Simulated and measured reflection spectra of the proposed composite nanostructure with a 20-nm-thick Au film between the nanodisk array and the bulk Al_2_O_3_ substrate.

Figure 1(a) demonstrates a schematic diagram of the proposed substrate-modulated composite nanostructure, where a triangular array of Au nanodisks is placed onto a thin Au film supported by a bulk Al_2_O_3_ dielectric substrate. Its top surface is immersed in a bulk aqueous solution of the RI of 1.33. The lower-right inset shows its cross-section view with the periodicity of the nanodisk array (*P*), the nanodisk’s diameter (*D*) and height (*t*), as well as the thickness of Au film (*d*) indicated. Figure 1(c) displays the simulated reflection spectrum of the composite nanostructure, depicted as the blue solid line. Obviously, two resonance dips are observed at 678 nm and 797 nm, marked as A and B, respectively. The corresponding experiment result is shown as the red solid line of Figure 1(c), two resonance dips appear at 651 nm and 815 nm. The scanning electron microscopy (SEM) images of the experimentally fabricated sample are shown in Figure 1(b). Based on the number of resonance dips and the shape of the reflection spectrum, the measured results are roughly consistent with the simulated one. However, some differences observed, including the wavelength positions, the depth and linewidth of the resonance dips. These discrepancies are attributed to several factors: firstly, the geometry parameters of the experimentally fabricated sample differ from the simulated one. Specifically, our experiment sample is manufactured by using the ultrathin AAO template transfer nanoimprinting, resulting in some inevitable flaws, such as quasi-periodic arrangement, random distribution of nanodisk’s diameter and morphology within a specific range, the edge defects of nanodisk, surface roughness, *etc*., which hasn’t been considered in our perfect simulation; Secondly, the permittivity of the experimental Au material slightly differs from that used in the simulation, especially for the imaginary part of the permittivity; Thirdly, an optical fiber collimator with a nonzero numerical aperture (NA) is used for the spectral measurements. The nonzero NA collimator cause the measured sample to be irradiated at oblique incidence angles, broadening the resonance linewidth and reducing the resonance depth.

**Figure 2:**
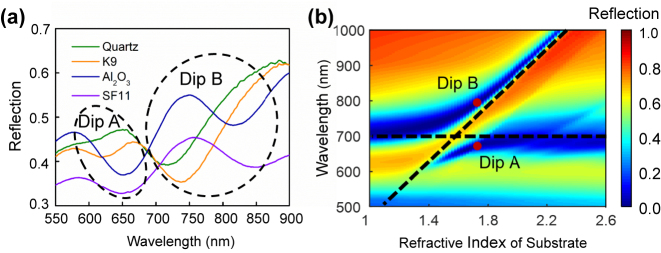
Dependence of (a) measured and (b) simulated reflection spectra of the composite nanostructure with a fixed 20-nm-thick Au film on a dielectric substrate with different RI. In the experiment, four types of dielectric substrates are considered, and their arrangement in order of increasing RI is quartz, K9, Al_2_O_3_, and SF11. Horizontal and inclined black dashed lines represent plasmon modes in the absence of interaction, respectively. These two hybridized plasmon modes in the composite nanostructure on an Al_2_O_3_ substrate using two solid red dots.

We first investigate the influence of the RI variation of the dielectric substrate on two resonance dips and its reflection spectrum, as shown in Figure 2(a). In the experiment, four common types of bulk dielectric substrates are selected in order of increasing RI, namely, quartz, K9, Al_2_O_3_, and SF11. Obviously, the dielectric substrate has a significant impact on the reflection spectrum. Concretely, when the low RI of quartz substrate is selected, Dip A is almost not stimulated. As the RI of the substrate increases, resonance Dip A first initially undergoes a redshift, accompanied by increased depth. When the dielectric substrate is switched to SF11, Dip A shows a slight redshift while its linewidth gets broadened, and its depth becomes shallower as compared to the Al_2_O_3_ substrate. Thereby, Dip A achieves the most superior properties with the Al_2_O_3_ substrate: relatively high depth and narrow linewidth. Furthermore, the increase in the RI of the substrate results in a continuous redshift of the wavelength of Dip B and has a weak effect on the depth and linewidth of Dip B. Compared to the variation of these two dips, Dip B is more susceptible to the RI of the dielectric substrate. Furthermore, Figure 2(b) shows the dependence of the simulated reflection spectrum on the successive increasing RI of the substrate from 1 to 2.6. The simulated results demonstrate the variation tendency of these two resonance dips with the RI of the substrate, which is nearly consistent with experiment one in Figure 2(a). Notably, the linewidth variation of Dip B in the simulation is slightly different from the experiment case, which may be caused by the inconsistent structural and material parameters. The optimized Al_2_O_3_ substrate is for further investigation due to both narrow linewidth and high depth of two resonance dips.

**Figure 3:**
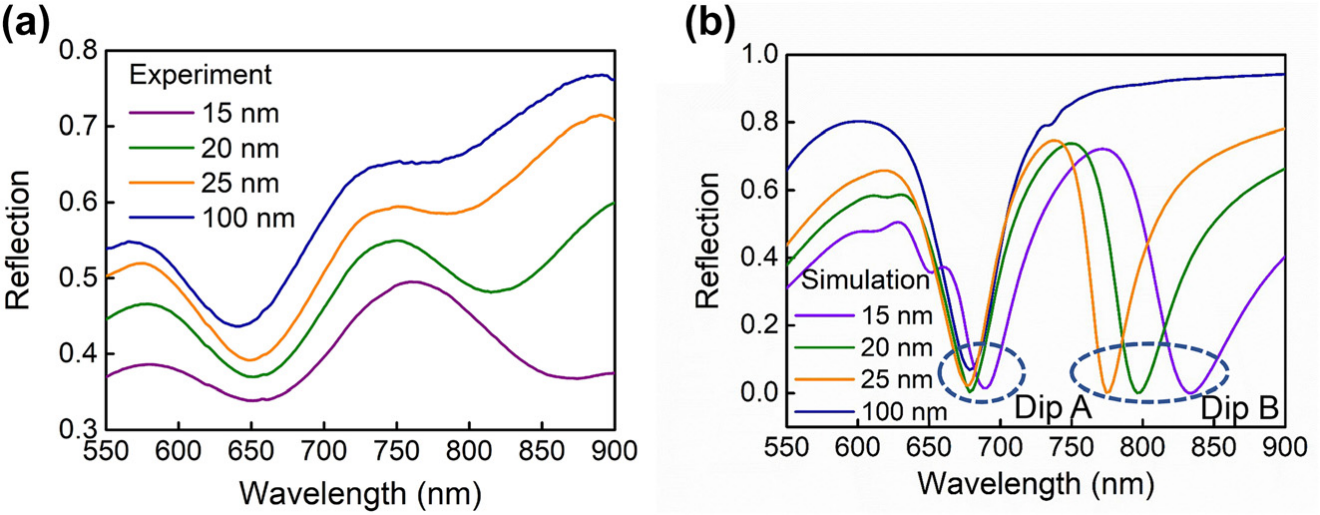
Dependence of (a) measured and (b) simulated reflection spectra of the composite nanostructure on the thickness *d* of the middle Au film between the top Au nanodisk array and the underlying dielectric substrate.

To further demonstrate the optical spectral adjustability of the proposed composite nanostructure, the influence of the thickness of the middle Au film on the reflection spectra is theoretically and experimentally investigated, as shown in Figure 3. We consider four thicknesses for the Au film *d*, that is, 15 nm, 20 nm, 25 nm, and 100 nm. It is observed that, as the thickness of the Au film increases, both resonance dips experience a blueshift, with Dip B showing a more distinct wavelength blueshift than Dip A in Figure 3(a). Meanwhile, the depth of Dip B in the experiment significantly declines and almost disappears when the Au film’s thickness approaches 100 nm in Figure 3(b). However, the depth of Dip A is somewhat enhanced during this process. This phenomenon occurs because the increased thickness of the Au film prevents the optical field from penetrating into the dielectric substrate. It is predicted that the appearance of Dip B is closely related to the dielectric substrate.

**Figure 4:**
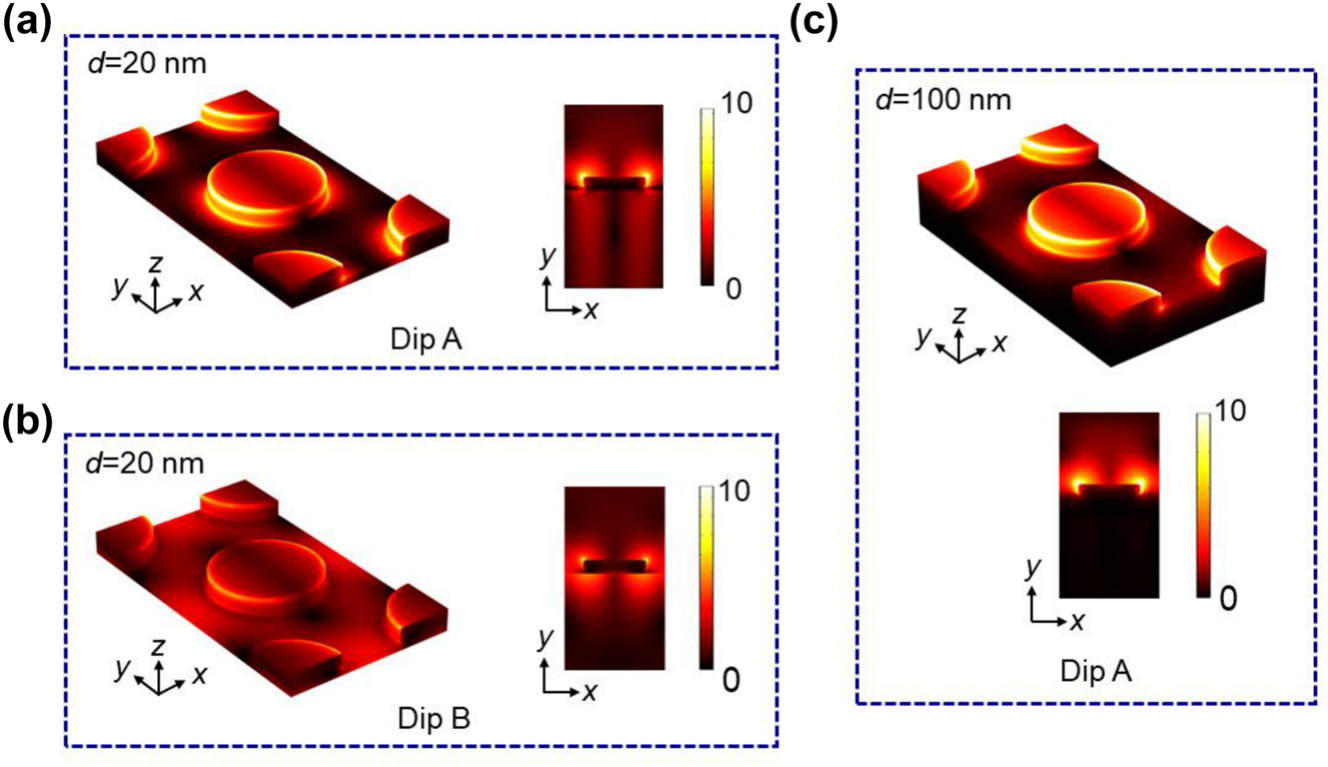
Spatially distributions of electric field amplitude of the composite nanostructure of Au nanodisk array/thin film. (a) Dip A and (b) Dip B with a 20-nm-thick Au film. As a direct comparison, Dip A with a 100-nm-thick Au film is given in (c).

To elaborate the generation mechanisms of two resonance dips, the electric field amplitude distributions for Dip A and Dip B with a fixed 20-nm-thick Au film are shown in Figure 4(a) and (b). For Dip A, the electric field is primarily localized at the two edges of the top surface of the nanodisk, forming localized surface plasmons (LSPs). Meanwhile, this field distribution is modulated by surface plasmon polaritons (SPPs) mode at the top surface generated by the array coupling, meaning that the adjacent nanodisk interact though the SPPs surface wave. Therefore, Dip A exhibits features of both LSPs and SPPs modes, also termed as collective plasmon mode in the reported literatures [35], [36]. Furthermore, it is also notable that a small amount of optical field passes through thin Au film then penetrates into the dielectric substrate. The resulting optical field is trapped at the Au film-substrate interface, forming SPPs at the bottom surface of Au film. Thus, the generation of Dip A is mainly determined by collective plasmons mode at the top surface and is perturbated by SPPs at the bottom surface of Au film.

In contrast, for Dip B, the SPPs mode at the bottom surface is greatly enhanced, the distribution of which is nearly consistent with that at the top surface except for the sharp rims of the nanodisk. Both collective plasmon mode and SPPs at the top and bottom surfaces of the composite structure contribute equally to the generation of Dip B. It is prominent to point out that SPPs at the bottom surface has no ability to be directly excited by the incidence light due to their wavevector mismatch. In this composite nanostructure, SPPs at the bottom surface is excited by the near-field coupling and hybridization of collective plasmon mode at the top surface. Notably, the generation of Dips A and B stems from mode hybridization and coupling of plasmon modes at the top and bottom surfaces. Referring back to Figure 2(b), collective plasmon mode and SPPs mode at the top and bottom surfaces in the absence of interaction are depicted by horizontal and inclined black dashed lines, respectively. It can be seen that these two modes in the proposed composite nanostructure interact and repel each other when the RI of substrate is 1.732, giving rise to two hybridized resonant modes, namely Dips A and B. When the RI of the substrate is either too large or too small, the hybridized coupling between them is almost negligible. Their interaction is limited to a narrow RI range of substrate. Therefore, Dips A and B in the proposed structure stem from the substrate-induced hybridization and coupling of collective plasmon mode and SPPs mode at the top and bottom surfaces.

As a direct comparison, Figure 4(c) demonstrates the distribution of the electric field amplitude of resonance Dip A when the middle Au film’s thickness is 100 nm. The unhybridized collective plasmon mode can only be excited at the top surface of the structure due to the opaque Au film. We notice that the electric field amplitude of the top surface of the unhybridized mode is stronger than that of individual hybridized mode. It is deduced that these two hybridized modes share the electric field intensity of top surface of the original mode.

**Figure 5:**
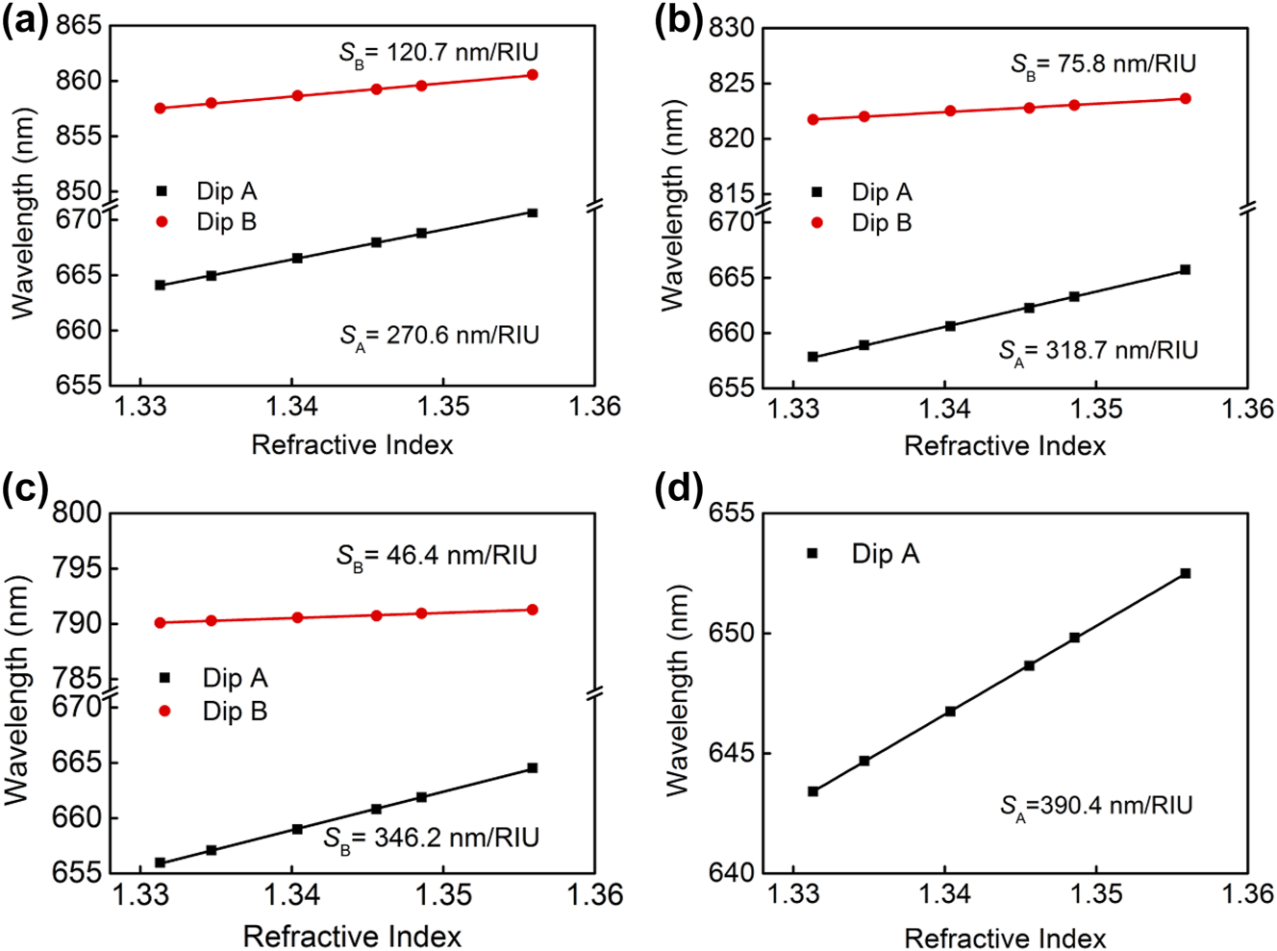
Experimentally measured wavelength positions of resonance modes Dip A and Dip B for different RIs of NaCl solutions as the superstrate for the middle Au film’s thickness of (a) 15 nm, (b) 20 nm, (c) 25 nm, and (d) 100 nm. The bulk RI sensitivity is obtained by linearly fitting the relationship between wavelength positions and the RIs of the superstrate. Note that only Dip A is considered for a 100-nm-thick Au film.

Next, we will further investigate the relationship of electric field amplitudes at the top surface before and after hybridization by investigating the bulk RI sensitivity and biomolecular detection of two resonance dips. Firstly, the bulk RI sensitivity of the composite nanostructure with the Au film thickness of 15 nm, 20 nm, 25 nm, and 100 nm are experimentally investigated, as shown in Figure 5. In the experiment, six concentrations of sodium chloride (NaCl) solutions are prepared with the RIs of 1.3327, 1.3347, 1.3404, 1.3456, 1.3486, and 1.3560. The bulk RI sensitivity of resonance dip is achieved by linearly fitting the relationship between wavelength positions and the RIs of NaCl solutions. The results of bulk RI sensitivities of Dips A and B and their summation for different thicknesses of the middle Au film are summarized in Table 1. As a result, bulk RI sensitivities of the composite nanostructure are calculated as *S*_A_ = 270.6 nm/RIU, *S*_B_ = 120.7 nm/RIU for a 15-nm-thick Au film, *S*_A_ = 318.7 nm/RIU, *S*_B_ = 75.8 nm/RIU for a 20-nm-thick Au film, *S*_A_ = 346.2 nm/RIU, *S*_B_ = 46.4 nm/RIU for a 25-nm-thick Au film, and *S*_A_ = 390.4 nm/RIU for a 100-nm-thick Au film, respectively. It is found that the bulk RI sensitivity of Dip A progressively increases with the increase of the middle Au film’s thickness. In contrast, the counterpart of Dip B is just opposite and continuously decreases. Additionally, the summation of bulk RI sensitivities for two resonance dips is 391.3 nm/RIU for a 15-nm-thick Au film, 394.5 nm/RIU for a 20-nm-thick Au film, 392.6 nm/RIU for a 25-nm-thick Au film, respectively, which roughly equals to the 390.4 nm/RIU for a 100-nm-thick Au film. The result of bulk RI sensitivity proposes good evidence for our viewpoint that these two hybridized modes in the composite structure share the electric field intensity at the entire top surface of the unhybridized mode, despite being excited at different wavelengths spaced far apart.

**Table 1:** Bulk RI sensitivity for different thicknesses of Au film with for the composite nanostructure.

The thickness of Au film	*S*_A_	*S*_B_	*S*_A_ + *S*_B_
15 nm	270.6 nm/RIU	120.7 nm/RIU	391.3 nm/RIU
20 nm	318.7 nm/RIU	75.8 nm/RIU	394.5 nm/RIU
25 nm	346.2 nm/RIU	46.4 nm/RIU	392.6 nm/RIU
100 nm	390.4 nm/RIU	–	390.4 nm/RIU

**Figure 6:**
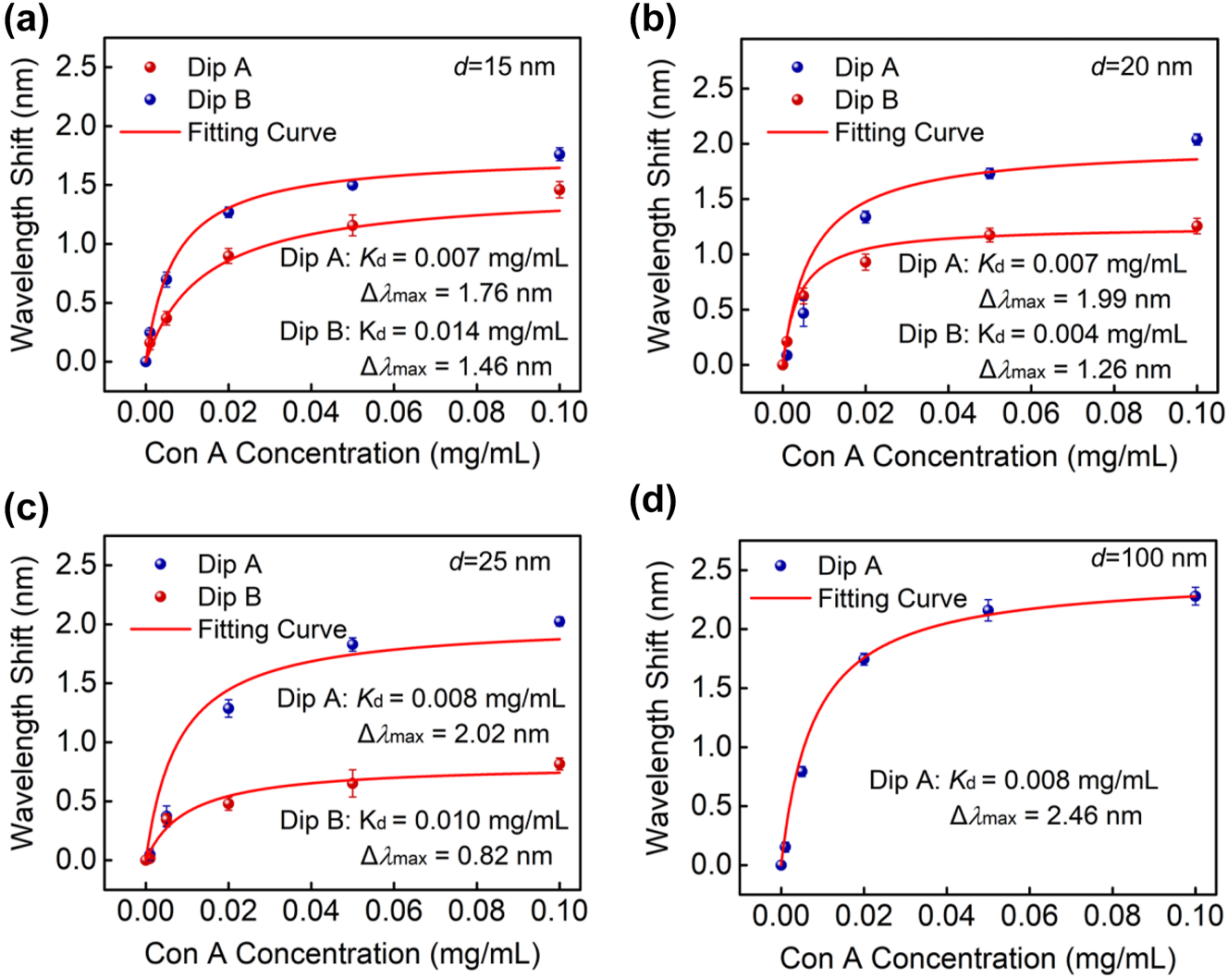
The wavelength shifts of resonance Dips A and B in response to different concentrations of protein molecular of Con A with the middle Au film’s thickness of (a) 15 nm, (b) 20 nm, (c) 25 nm, and (d) 100 nm. The red curves are fitted to the measured results using the Langmuir isotherm. The error bars represent the standard deviation for three repeated measurements.

The above bulk RI sensitivity only validates the variation of the optical field in the top half-space of the composite nanostructure, its near-fields within a nanometer-scale range also need to be considered. Here, we consider the near-field variations of two resonance dips within a nanometer-scale range by measuring wavelength shift amount at different concentrations of protein molecular of Con A by using the specific binding of RNase B and Con A. Molecular self-assembly, surface activation, its restoration on the surface of the composite nanostructure, and specific measurement process for protein Con A have been given in our previous literature [33]. Different concentrations of molecular Con A from 0.001 mg/mL, 0.005 mg/mL, 0.02 mg/mL, 0.05 mg/mL to 0.1 mg/mL are measured using the proposed composite nanostructure with the Au film’s thickness of 15 nm, 20 nm, 25 nm, and 100 nm. The measured results are shown in Figure 6, where the relationship between the wavelength shift and Con A concentration is fitted using the well-known Langmuir isotherm (red solid line) [37] It is observed that wavelength shifts of all resonance dips initially rise quickly, then gradually approach saturation with increasing the Con A concentration, indicating their local near-field characteristics of two resonance dips. Furthermore, the biomolecular detection sensitivity of Dip A is superior to that in Dip B, which is consistent with the bulk RI sensitivity. According to the fitting results, the maximum wavelength shift Δ*λ* induced by the protein molecule of Con A for resonance Dips A and B, and their summation for different thicknesses of Au film are summarized in Table 2. As a result, the maximum wavelength shift Δ*λ* are calculated as Δ*λ*_A_ = 1.76 nm, Δ*λ*_B_ = 1.46 nm for a 15-nm-thick Au film, Δ*λ*_A_ = 1.99 nm, Δ*λ*_B_ = 1.26 nm for a 20-nm-thick Au film, *S*_A_ = 2.02 nm, *S*_B_ = 0.82 nm for a 25-nm-thick Au film, and *S*_A_ = 2.46 nm for a 100-nm-thick Au film, respectively. It is found that the maximum wavelength shift Δ*λ* of Dip A gradually increases with the increase of the middle Au film’s thickness, while the counterpart of Dip B is just opposite and continuously decreases. Furthermore, the summation of the maximum wavelength shifts for two resonance dips is 3.22 nm for a 15-nm-thick Au film, 3.25 nm for a 20-nm-thick Au film, and 2.84 nm for a 25-nm-thick Au film, respectively. The detection of protein molecules gives a somewhat different conclusion that these two hybridized modes in the composite structure, to a certain extent, share the electric field intensity at the top surface before the unhybridized mode. It needs to be pointed out that the molecular detection sensitivity of each hybrid mode, Dip A and Dip B, is lower than the unhybridized mode, but their summation of the detection sensitivity is higher. It can be deduced that the summation of near-fields within a nanometer-scale range at the top surface for two hybridized modes is larger than that of the unhybridized mode, which further validates the ability of mode hybridization to redistribute near field within a nanometer-scale range.

**Table 2:** The maximum wavelength shift Δ*λ* induced by the protein molecule Con A with different thicknesses of Au film for the composite nanostructure.

The thickness of Au film	Δ*λ*_A_	Δ*λ*_B_	Δ*λ*_A_ + Δ*λ*_B_
15 nm	1.76 nm	1.46 nm	3.22 nm
20 nm	1.99 nm	1.26 nm	3.25 nm
25 nm	2.02 nm	0.82 nm	2.84 nm
100 nm	2.46 nm	–	2.46 nm

## 4 Conclusions

In summary, we have theoretically and experimentally demonstrated a plasmonic composite nanostructure of an Au nanodisk array and a thin Au film supported by a dielectric substrate. This structure achieves two resonance modes originating from the hybridization coupling of plasmon modes at the upper and lower surfaces. These two hybridized modes can be simultaneously modulated by both the RI of the dielectric substrate and the thickness of translucent Au film. Furthermore, the relationship between near-field amplitudes of two hybrid modes and the unhybridized mode in the nanodisk array on the opaque Au film at both top half-space and a nanometer-scale range is further verified by both bulk RI sensitivity and molecular detection capability. This work provides a detailed investigation on the substrate-induced hybridization of plasmon modes in the composite nanostructure, especially the optical field distribution of resonance modes before and after hybridization. This finding offers some new insights for their further practical application, enhancing our understanding of the behavior of hybridized plasmon modes in such structures.

## References

[1] Yang K., Yao X., Liu B., Ren B. (2021). Metallic plasmonic array structures: principles, fabrications, properties, and applications. *Adv. Mater.*.

[2] Huang L. (2021). One-step rapid quantification of SARS-CoV-2 virus particles via low-cost nanoplasmonic sensors in generic microplate reader and point-of-care device. *Biosens. Bioelectron*..

[3] Tobing L. Y. M. (2021). Interplays of dipole and charge-transfer-plasmon modes in capacitively and conductively coupled dimer with high aspect ratio nanogaps. *Adv. Opt. Mater.*.

[4] Wang B. (2021). High-Q plasmonic resonances: fundamentals and applications. *Adv. Opt. Mater.*.

[5] Tanaka T., Yano T., Kato R. (2022). Nanostructure-enhanced infrared spectroscopy. *Nanophotonics*.

[6] Xu T., Geng Z. (2021). Strategies to improve performances of LSPR biosensing: structure, materials, and interface modification. *Biosens. Bioelectron*..

[7] Nan J. (2020). Ultrahigh-sensitivity sandwiched plasmon ruler for label-free clinical diagnosis. *Adv. Mater.*.

[8] Chen L. (2023). Tunable layered gold nanochips for high sensitivity and uniformity in SERS detection. *J. Phys. Chem. C*.

[9] Park J. H. (2020). Symmetry-breaking-induced plasmonic exceptional points and nanoscale sensing. *Nat. Phys.*.

[10] Bardhan R., Mukherjee S., Mirin N. A., Levit S. D., Nordlander P., Halas N. J. (2010). Nanosphere-in-a-nanoshell: a simple nanomatryushka. *J. Phys. Chem. C*.

[11] Zhang S., Bao K., Halas N. J., Xu H., Nordlander P. (2011). Substrate-induced Fano resonances of a plasmonic: nanocube: a route to increased-sensitivity localized surface plasmon resonance sensors revealed. *Nano Lett.*.

[12] Braïk M. (2023). Hybridization of surface lattice modes: towards plasmonic metasurfaces with high flexible tunability. *Nanophotonics*.

[13] Du X., Xiong L., Zhao X., Chen S., Shi J., Li G. (2022). Dual-band bound states in the continuum based on hybridization of surface lattice resonances. *Nanophotonics*.

[14] Kotlarek D. (2020). Actuated plasmonic nanohole arrays for sensing and optical spectroscopy applications. *Nanoscale*.

[15] Mohammadi R., Ochs M., Andrieu-Brunsen A., Vogel N. (2019). Effect of asymmetry on plasmon hybridization and sensing capacities of hole-disk arrays. *J. Phys. Chem. C*.

[16] Ding F., Yang Y. Q., Deshpande R. A., Bozhevolnyi S. I. (2018). A review of gap-surface plasmon metasurfaces: fundamentals and applications. *Nanophotonics*.

[17] Chikkaraddy R., Baumberg J. J. (2021). Accessing plasmonic hotspots using nanoparticle-on-foil constructs. *ACS Photonics*.

[18] Baumberg J. J., Aizpurua J., Mikkelsen M. H., Smith D. R. (2019). Extreme nanophotonics from ultrathin metallic gaps. *Nat. Mater.*.

[19] Wang Z. X. (2023). Effect of mirror quality on optical response of nanoparticle-on-mirror plasmonic nanocavities. *Adv. Opt. Mater.*.

[20] Kandil S. M., Eshrah I. A., El Babli I. S., Badawi A. H. (2016). Plasmon hybridization in split ring nanosandwich for refractive index sensing-Numerical Investigation. *Opt. Express*.

[21] Kuo C. (2022). Sensitive oligonucleotide detection using resonant coupling between Fano resonance and image dipoles of gold nanoparticles. *ACS Appl. Mater. Interfaces*.

[22] Cetin A. E., Altug H. (2012). Fano resonant ring/disk plasmonic nanocavities on conducting substrates for advanced biosensing. *ACS Nano*.

[23] Chen J., Gan F., Wang Y., Li G. (2018). Plasmonic sensing and modulation based on Fano resonances. *Adv. Opt. Mater.*.

[24] Ai B., Song C., Bradley L., Zhao Y. (2018). Strong Fano resonance excited in an array of nanoparticle-in-ring nanostructures for dual plasmonic sensor applications. *J. Phys. Chem. C*.

[25] Feng T., Xiang J., Liu C., Geng Z. (2023). Gold nano-double-ring array sensor based on Fano resonance. *Opt. Commun*..

[26] Wu T., Yang S., Tan W., Li X. (2016). Tunable localized hybrid plasmon modes and Fano resonances in Au core-semishell. *Plasmonics*.

[27] Li A., Wang X., Guo L., Li S. (2019). Tunable subradiant mode in free-standing metallic nanohole arrays for high-performance plasmofluidic sensing. *J. Phys. Chem. C*.

[28] Cetin A. E., Etezadi D., Galarreta B. C., Busson M. P., Eksioglu Y., Altug H. (2015). Plasmonic nanohole arrays on a robust hybrid substrate for highly sensitive label-free biosensing. *ACS Photonics*.

[29] Movsesyan A. (2021). Hybridization and dehybridization of plasmonic modes. *J. Phys. Chem. C*.

[30] Cherqui C., Li G., Busch J. A., Quillin S. C., Camden J. P., Masiello D. J. (2018). Multipolar nanocube plasmon mode-mixing in finite substrates. *J. Phys. Chem. Lett.*.

[31] Félidj N. (2002). Enhanced substrate-induced coupling in two-dimensional gold nanoparticle arrays. *Phys. Rev. B*.

[32] Vala M., Ertsgaard C. T., Wittenberg N. J., Oh S. (2019). Plasmonic sensing on symmetric nanohole arrays supporting high-Q hybrid modes and reflection geometry. *ACS Sens.*.

[33] Liang Y., Cui W., Li L., Yu Z., Peng W., Xu T. (2019). Large‐scale plasmonic nanodisk structures for a high sensitivity biosensing platform fabricated by transfer nanoprinting. *Adv. Opt. Mater.*.

[34] Johnson P. B., Christy R. W. (1972). Optical constants of the noble metals. *Phys. Rev. B*.

[35] Gantzounis G., Stefanou N., Papanikolaou N. (2008). Optical properties of periodic structures of metallic nanodisks. *Phys. Rev. B*.

[36] Cesario J., Quidant R., Badenes G., Enoch S. (2005). Electromagnetic coupling between a metal nanoparticle grating and a metallic surface. *Opt. Lett.*.

[37] Guo Q. (2014). Silicon-on-glass graphene-functionalized leaky cavity mode nanophotonic biosensor. *ACS Photonics*.

